# A Demonstration of the Nerves of the Human Body

**Published:** 1834-10-01

**Authors:** 


*A Demonstration of the Nerves of the Human Body.* By Joseph Swan.—London, 1834. 4to. pp. 98 and lxxxii. Twenty-five Plates.

It is related that, when Voltaire was asked why he did not write critical observations on Racine, as he had done on Corneille, he answered, "*C'est deja fait; vous n'avez qu'à écrire en bas de chaque page, beau, pathétique, sublime, harmonieux.*" Mr. Swan's Plates of the Nerves place us in a like difficulty, and render criticism impossible: we have nothing to do but to admire and learn. The present work contains copies of the plates in the folio edition, and, though the size is smaller, the engravings are exquisitely distinct, even to the minutest fibrils. The reduction of size, too, has been accompanied by even more than a corresponding reduction in price, so that every friend to science may not only hope, but expect, that the book will be in everybody's hands.

The following passage, which details some interesting points in neurology, will show the refined accuracy of Mr. Swan's demonstrations.

" *The Third Trunk of the Threefold, or Fifth Pair of Nerves.*

" It passes through the oval foramen of the sphenoid bone, and divides into five principal nerves. But, before describing these, it will be necessary to speak of the anterior portion of the fifth accompanying it.

" The anterior portion appears to consist of four principal fibrils; the most anterior passes down, and, after becoming connected with the Gasserian ganglion, goes to the part of the branch chiefly terminating in the temporal muscle; the second, towards its most inferior part, likewise gives a filament to the same deep temporal branch ; the rest of the second fibril and the third are given to the buccal nerve, and the fourth joins the gustatory.

" The deep temporal nerve passes externally, and gives a branch to be joined with the first and part of the second fibril of the anterior portion, and then distributed to the temporal muscle; it communicates with the branch of the facial nerve joining the temporal branch of the malar by filaments which pierce the temporal muscle; the rest of the nerve passes just anterior to the condyle of the lower jaw, and proceeds downwards, to terminate in the masseter muscle, after giving filaments to the capsular ligament of the lower jaw: sometimes this joint is supplied by the superficial temporal.

" The superficial temporal nerve is formed of two branches: it passes behind the condyloid process of the lower jaw; it sends a filament on the external carotid artery, to anastomose with filaments from the sympathetic, and with one from the inferior dental passing on the internal maxillary artery; it communicates with the facial nerve by three branches, and gives filaments to the membrane lining the external auditory meatus, and the skin at the anterior part of the ear, and mounts up to the temple, to terminate on the skin of this part.

" The inferior dental nerve, the continuation of which forms the inferior maxillary, descends between the two pterygoid muscles, and sends a filament on the beginning of the internal maxillary artery to communicate with the filaments of the sympathetic nerve, ramifying on the internal carotid artery, also with the other branches from the spheno-palatine ganglion, passing on the internal maxillary artery; it sends a branch downwards for some distance in a groove in the lower jaw, to pass, just before the anterior corner of the submaxillary gland, to which it gives a small branch, and then between the mylo-hyoideal muscle and the maxillary portion of the digastric, and divides and terminates in these muscles. The inferior dental nerves, accompanied by the dental artery, passes into the aperture at the superior and posterior part of the lower jaw, and, almost as soon as it has entered this, sends a branch downwards, which gives a filament to the posterior fang of the second molar tooth, and passes forward round the superior part of the same fang, to terminate in the cancellated structure. It then proceeds just beneath the fangs of the teeth, and gives a filament to each fang of the molar, and the second bicuspidated; it sends a branch forward to give a filament to the first bicuspidated, the cuspidated, and incisive teeth, and then passes out at the foramen near the chin, and, after communicating with the facial nerve, terminates in the buccal glands, the muscles, and skin of the lower lip and chin.

"The buccal nerve appears to be principally formed of the third fibril, and part of the second of the anterior portion of the fifth; it gives branches to the temporal and external pterygoid muscles, and then passes close to the inner surface of the coronoid process of the lower jaw; in its course some filaments are distributed to the membrane lining the mouth, and, after emerging from behind the masseter muscle, it communicates with the facial nerve, sends filaments on the facial artery to join others from the sympathetic nerve, and then terminates in the buccal glands, the muscles, and skin at the side of the mouth. Near the part whence the buccal nerve arises, filaments are given to the circumflex muscle of the palate.

"The gustatory is at first so connected with the inferior dental, as to appear part of the same nerve. It receives the fourth fibril of the anterior portion, and is joined by the chord of the tympanum just after it has separated from the inferior dental; it then communicates with this nerve; it passes at first between the internal and external pterygoid muscles, and to the former of these gives a filament; it then passes just behind the last molar tooth, underneath the membrane of the mouth, giving filaments to this, and winding over the superior edge of the submaxillary gland, expands into a broad and flat nerve, which frequently assumes a ganglionic appearance; it gives many filaments to the submaxillary and sublingual glands, and to the membrane situated between the outer edge of the tongue and lower jaw; it communicates with the ninth nerve, and, in passing over the insertion of the hyo-glossal muscle, divides into several branches, which proceed by the exterior side of the lingual artery between the insertion of the genio-hyo-glossal and lingual muscles, then subdivide into smaller branches, pass through the delicate muscular fibres composing the superficial part of this organ, and terminate in a villous form on two-thirds of the anterior portion of the external surface. It is difficult to decide whether some filaments, in penetrating through this fine muscular structure of the tongue, do not partly terminate on it.

"The chord of the tympanum gives filaments to the surrounding membranous structure, and then enters a canal by the fissure of Glaser; it gives filaments in its course, and particularly about the membrane of the tympanum, and the laxator and tensor muscles; it passes over the handle of the hammer to be joined to the facial nerve, just before this emerges from the stylo-mastoid foramen. It is supposed that the chord of the tympanum does not unite with the gustatory nerve, but passes in mere contact with this; but if a preparation that has been kept in spirits be carefully examined with a magnifying-glass, and at the same time an attempt be made to disunite these nerves, it will be found that the filaments of both are intermixed, and cannot be separated without violence." (P. 41.)

